# Endogenous γ-Aminobutyric Acid Accumulation Enhances Salinity Tolerance in Rice

**DOI:** 10.3390/plants13192750

**Published:** 2024-09-30

**Authors:** Mingjia Chen, Changhua Zhu, Hui Zhang, Siheng Chen, Xi Wang, Lijun Gan

**Affiliations:** College of Life Sciences, Nanjing Agricultural University, Nanjing 210095, China; mjchen@njau.edu.cn (M.C.); zch@njau.edu.cn (C.Z.); 2021116008@stu.njau.edu.cn (H.Z.); sihengchen96@gmail.com (S.C.); 2022816136@stu.njau.edu.cn (X.W.)

**Keywords:** γ-aminobutyric acid, rice, salt stress

## Abstract

Rice is an important food crop worldwide but is usually susceptible to saline stress. When grown on soil with excessive salt, rice plants experience osmotic, ionic, and oxidative stresses that adversely affect growth performance. γ-Aminobutyric acid (GABA) is a nonproteinogenic amino acid that plays an important role in the metabolic activities of organisms. Glutamate decarboxylase (GAD) is the rate-limiting enzyme in GABA metabolism. Here, we genetically modified rice GAD by overexpression or CRISPR-mediated genome editing. These lines, named *gad3-ox1* and *gad3-ox2* or *gad1/3-ko*, were used to explore the effects of endogenous GABA accumulation on salt tolerance in rice. Both the *gad3-ox1* and *gad3-ox2* lines exhibited significant accumulation of the GABA content, whereas the *gad1/3-ko* line presented a reduced GABA content in vivo. Notably, the two overexpression lines were markedly resistant to salt stress compared with the wild-type and knockout lines. Furthermore, our results demonstrated that endogenous GABA accumulation in the *gad3-ox1* and *gad3-ox2* lines increased the contents of antioxidant substances and osmotic regulators, decreased the content of membrane lipid peroxidation products and the Na^+^ content, and resulted in strong tolerance to salt stress. Together, these data provide a theoretical basis for cultivating rice varieties with strong salt tolerance.

## 1. Introduction

Rice (*Oryza sativa* L.) is an important crop that provides food for more than half of the world’s population. Approximately 90% of global rice cultivation is performed in Asia, with China accounting for 30% [1,2]. According to statistics, approximately 30% of the rice cultivation fields worldwide contain saline soil [3]. Rice is a salt-sensitive plant, especially during the seedling and reproductive stages [4,5,6,7]. Saline soil strongly affects rice growth performance by reducing photosynthesis, leaf growth, and biomass production and causing low grain yield [8,9]. Owing to climate change and improper fertilisation and irrigation by humans, the saline soil area is still expanding, and soil salinisation has become a serious crisis [10,11,12].

The damage caused by salt stress in plants can occur in two stages: rapid osmotic stress and slow ion toxicity [4,13]. In the initial stage, due to the increase in the Na^+^ concentration in the soil, the soil water potential decreases, making it difficult for plants to absorb water. In response to this stress, plants usually synthesise osmolytes, such as proline and soluble sugars, to increase their ability to maintain cell volume and turgor pressure [14]. Metabolomic profiling analysis of Arabidopsis, rice, and lotus plants revealed that the metabolic balance of amino acids and organic acids is affected by salt stress [15].

During the second stage, that is, the ion toxicity stage, plants absorb and accumulate more Na^+^. Owing to the molecular similarity of Na^+^ and K^+^, excessive absorption of Na^+^ inhibits the uptake of K^+^, leading to an intracellular ion imbalance [16]. Whether plants can maintain a low Na^+^/K^+^ ratio is an important indicator of their salt resistance. Multiple ion channels and transporters in plants play crucial roles in maintaining cellular Na^+^/K^+^ homeostasis. Under salt stress, excessive accumulation of Na^+^ leads to elevated levels of apoplastic reactive oxygen species (ROS), such as hydrogen peroxide (H_2_O_2_), superoxide radicals (O_2_^•−^) and hydroxyl radicals (^•^OH), which disrupt redox homeostasis and cause oxidative damage to plant cells by attacking biological macromolecules and causing membrane injury [17,18]. Plants eliminate excessive ROS through antioxidant systems. Under salt stress, plants usually activate their antioxidant enzymes, such as superoxide dismutase (SOD) and peroxidase (POD), thereby eliminating the excess ROS and enhancing plant salt resistance [9,19,20].

γ-Aminobutyric acid (GABA) is a nonprotein amino acid widely distributed in prokaryotes and eukaryotes [21]. In animals, GABA is an inhibitor of neurotransmitters that mediates the response of animals to stress by binding to GABA receptors. In plants, GABA was first discovered in potato tubers, after which cellular GABA metabolism and functions were gradually revealed. The metabolism of GABA in plants is mediated mainly by the GABA shunt pathway [22,23,24,25,26]. In the cytoplasm, glutamate decarboxylase (GAD) catalyses the conversion of glutamate to GABA, which is then transferred to mitochondria, where it is further metabolised to succinic acid by GABA transaminase (GABA-T) and succinate semialdehyde dehydrogenase (SSADH) and enters the TCA cycle [24,25]. GABA, as a metabolite, participates in pH regulation, the regulation of redox status and the maintenance of the carbon: nitrogen balance. GABA also acts as a signalling molecule to regulate pollen tube growth, stomatal movement and the response to stress [26,27,28,29,30].

GAD-mediated GABA biosynthesis is involved in the response of plants to various stresses. For example, under salt stress, the expression level of the *SlGAD1-3* gene in tomatoes increases, and the activity of GAD also significantly increases. GAD-modulated synthesis of GABA regulates drought and salt tolerance in Arabidopsis [31,32,33]. Exogenous GABA treatment can also significantly increase plant resistance to stress, such as salt, drought, and hypoxia stress [33,34]. Under drought conditions, exogenous GABA treatment increased the osmotic regulation and membrane integrity of white clover, thereby improving its drought resistance [35]. Under salt stress, exogenous GABA alleviated oxidative injury, reduced Na^+^ accumulation, and improved salt resistance in tomatoes [32].

In total, the rice plant possesses five GAD genes, including a gene without expression in any plant tissues [36]. Here, OsGAD3 was employed to construct *GAD*-overexpressing (*gad3-ox1* and *gad3-ox2*) and GAD knockout (*gad1/3-ko*) lines due to its strong expression in rice [37,38]. Compared with those in the wild type (WT), the GABA contents in *gad3-ox1* and *gad3-ox2* increased, whereas the GABA content in *gad1/3-ko* decreased. Na^+^ and K^+^ ion homeostasis and ROS production were also altered in different genotypes upon salt treatment. In addition, GABA accumulation increased the proline content in rice under salt treatment. By comparing the responses of the WT, *gad3-ox1*, *gad3-ox2* and *gad1/3-ko* lines to salt stress, we elucidated the mechanisms underlying the role of GABA in salt tolerance in rice.

## 2. Results

### 2.1. GABA Accumulation Enhanced Salt Tolerance in Rice

**Figure 1:**
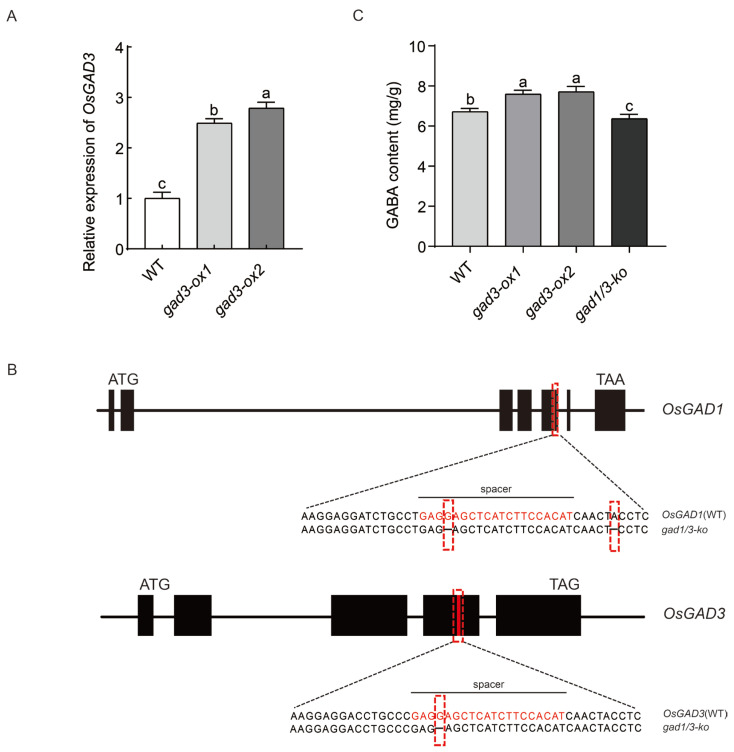
Generation and analysis of genome-edited plants. (**A**) Expression of the *OsGAD3* gene in WT, *gad3-ox1* and *gad3-ox2*. Error bars are SD (n = 3 biological replicates). (**B**) Schematic diagram of the *OsGAD1* and *OsGAD3* gene structure and gRNA target site for CRISPR/Cas9-mediated mutagenesis of *OsGAD1* and *OsGAD3*. The black rectangles represent the exon regions. The spacer location is marked in red—nucleotide sequence alignment of WT *OsGAD1* and *OsGAD3* and the *gad1/3-ko* mutants. The sequence marked in red text under the black line indicates the spacer sequence. (**C**) GABA content in germinating seeds of rice of each strain. The *OsActin* was selected as an internal control to normalise data. Experiments were repeated three times with similar results. Error bars are SD (n = 3 biological replicates). Different letters indicate significant differences at *p* < 0.05.

Exogenous GABA treatment can alleviate salt toxicity in various plants [29]. To investigate the mechanism underlying the role of GABA in salt tolerance, we constructed the *GAD3*-overexpressing lines *gad3-ox1* and *gad3-ox2* [39] and the CRISPR deletion mutant *gad1/3-ko* line (Figure 1A,B). Analysis of the endogenous GABA content revealed that, compared with those in the WT plants, the GABA concentrations in the *gad3-ox1* and *gad3-ox2* plants increased by 13.02% and 14.69%, whereas the GABA content in the *gad1/3-ko* plants decreased by 5.25% (Figure 1C).

**Figure 2:**
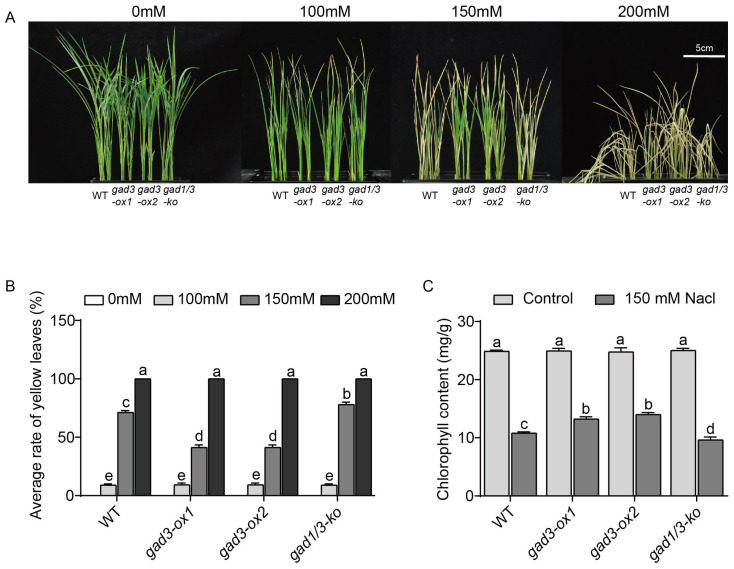
Growth status and chlorophyll content of each strain after 7 days of treatment with different concentrations of NaCl. (**A**) Phenotype. (**B**) Average percentage of yellow leaves. (**C**) Chlorophyll content. The error bars represent the SDs (n = 3 biological replicates). Different letters indicate significant differences at *p* < 0.05. Scale bar = 5 cm.

Salinity stress usually severely impedes plant growth [4,13]. To investigate the role of GABA content in rice under salinity stress, three-leaf-stage seedlings of *gad3-ox1*, *gad3-ox2*, *gad1/3-ko* and WT plants were exposed to different concentrations of NaCl for 7 days. The results revealed that 100 mM NaCl had a minor effect on growth but had no effect on the average percentage of yellow leaves in seedlings of the different rice lines. However, 150 mM NaCl caused prominent growth damage, such as leaf chlorosis, wilting and rolling (Figure 2A). The average percentage of yellow leaves of the WT, *gad3-ox1*, *gad3-ox2* and *gad1/3-ko* plants exposed to 150 mM NaCl was 71.33%, 41.33%, 41.33% and 78%, respectively. Compared with the WT plants, the *gad3-ox1* and *gad3-ox2* plants presented better growth performance, whereas the *gad1/3-ko* plants presented worse growth performance (Figure 2). Additionally, the growth of all the genotypes was strongly inhibited upon 200 mM NaCl treatment, e.g., the leaves were completely yellow and withered (Figure 2A). These results suggested that different doses of endogenous GABA in rice resulted in different resistance to salinity.

To investigate the effect of the GABA content on salinity stress, seedlings grown under 150 mM NaCl were chosen for further analyses. Salt stress often causes chlorophyll degradation and leaf yellowing, thus affecting plant photosynthesis and biomass. After 150 mM NaCl treatment for 7 days, some of the leaves from the WT plants presented yellow, curled and dry tips, whereas most of the leaves from the *gad1/3-ko* plants presented yellow colouration with completely curled and dry tips (Figure 2A). Interestingly, the increased accumulation of GABA in vivo, which was detected in the two overexpression lines (Figure 1A), could partially rescue such salt stress-induced phenotypic alterations (Figure 2). As shown in Figure 2C, in comparison with the control, salt stress decreased the concentration of total chlorophyll in each seedling. The concentrations of chlorophyll in the WT, *gad3-ox1*, *gad3-ox2* and *gad1/3-ko* plants decreased by 56.59%, 46.93%, 43.49% and 61.54%, respectively. In each treatment group, the total chlorophyll content in the two overexpression lines decreased less than that in the *gad1/3-ko* or WT seedlings, suggesting that GABA accumulation enhanced salt tolerance in rice.

### 2.2. Salt Treatment Induced GABA Accumulation in Rice Seedlings

**Figure 3:**
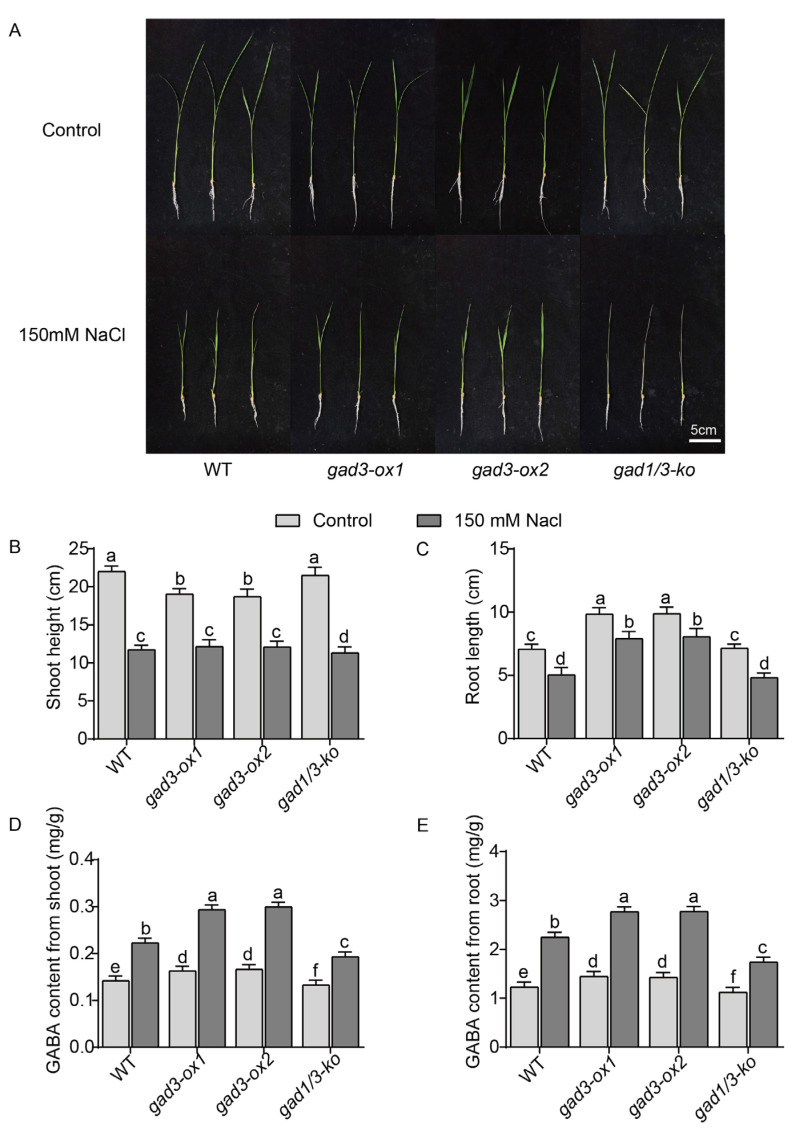
Growth status and GABA content of each strain after 7 days of 150 mM NaCl treatment. (**A**) Phenotype. (**B**) Shoot height. (**C**) Root length. (**D**) GABA content in shoots. (**E**) GABA content in roots. The error bars represent SDs (n = 3 biological replicates). Different letters indicate significant differences at *p* < 0.05. Scale bar = 5 cm.

To test whether the GABA content affects the salt stress response in young rice plants, we grew 21-day-old WT, *gad3-ox1*, *gad3-ox2*, and *gad1/3-ko* seedlings on 1/2 Kimura B nutrient solution supplemented with 150 mM NaCl for 7 days. NaCl treatment significantly inhibited the shoot growth of the WT, *gad3-ox1*, *gad3-ox2* and *gad1/3-ko* plants (Figure 3A,B). Compared with those of plants grown under normal conditions, the shoot heights of the WT, *gad3-ox1*, *gad3-ox2*, and *gad1/3-ko* plants exposed to salt stress decreased by 46.74%, 36.16%, 35.28% and 47.21%, respectively. The root lengths of the WT, *gad3-ox1*, *gad3-ox2*, and *gad1/3-ko* plants also decreased by 28.82%, 19.82%, 18.43% and 32.70%, respectively, compared with those of the seedlings grown under the salt treatment Furthermore, increased GABA content in vivo caused a reduction in rice seedling growth (Figure 3A–C), which was also confirmed by another study [40]. Notably, GABA accumulation decreased shoot height but increased root length, but these effects were compromised when the overexpression lines were subjected to salt treatment (Figure 3B,C). These results suggested that a relatively high GABA content reduced rice growth performance but improved salt tolerance.

To investigate the influence of salt treatment on GABA biosynthesis in rice, we also compared the GABA contents of WT, *gad3-ox1*, *gad3-ox2* and *gad1/3-ko* rice plants grown with and without 150 mM NaCl supplementation. The results indicated that the GABA content in the shoot samples from the WT, *gad3-ox1*, *gad3-ox2*, and *gad1/3-ko* rice seedlings increased 1.57-, 1.80-, 1.80-, and 1.45-fold, respectively, under salt treatment (Figure 3D). Interestingly, such salt stress-induced GABA accumulation in both overexpression lines was significantly greater than that in the WT and *gad1/3-ko* lines. A similar effect was observed in the root samples (Figure 3E). Taken together, these results demonstrated that a high endogenous GABA content can alleviate salt toxicity and, in turn, salt treatment can induce GABA accumulation in rice plants.

### 2.3. High Endogenous GABA Concentrations Altered Na^+^ and K^+^ Ion Homeostasis under Salinity Treatment

**Figure 4:**
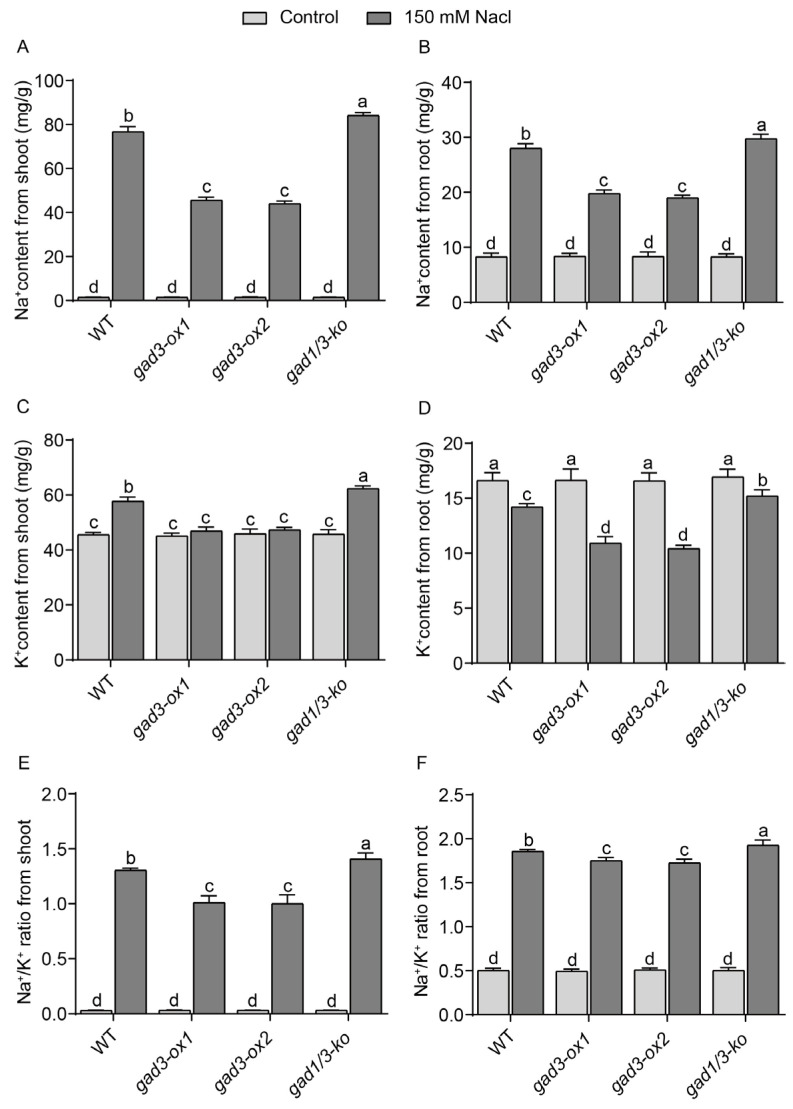
Na^+^ content, K^+^ content and Na^+^/K^+^ ratio of each strain after 7 days of 150 mM NaCl treatment. (**A**) Na^+^ content in shoots. (**B**) Na^+^ content in roots. (**C**) K^+^ content in shoots. (**D**) K^+^ content in the roots. (**E**) Na^+^/K^+^ ratio from shoot. (**F**) Na^+^/K^+^ ratio from root. The error bars represent SDs (n = 3 biological replicates). Different letters indicate significant differences at *p* < 0.05.

Salt stress can cause the accumulation of Na^+^ in rice plants and lead to ion imbalance. To maintain ion homeostasis, plants usually reduce the accumulation of Na^+^ and maintain a low Na^+^/K^+^ ratio, which mitigates the damage caused by Na^+^ toxicity [4,41]. As shown in Figure 4A,B, the Na^+^ levels in all the genotypes were very low when they were grown under normal conditions. However, the ion contents were markedly increased when the seedlings were grown under the 150 mM NaCl treatment. For each genotype grown under salt treatment, the increase in the Na^+^ content in the shoots or roots of the two overexpression lines was significantly lower than that in the shoots or roots of the WT and *gad1/3-ko* lines (Figure 4A,B), suggesting that GABA accumulation in vivo can decrease the Na^+^ level in seedlings grown under salt treatment. Specifically, the Na^+^ contents in the roots of the WT, *gad3-ox1*, *gad3-ox2*, and *gad1/3-ko* plants increased 3.38-, 2.36-, 2.27- and 3.59-fold, respectively, under salt treatment, which was consistent with the trend for the Na^+^ content in the roots. However, the increase in Na^+^ levels in the roots of each genotype was much lower than that in the shoots.

In addition, there was no difference in the K^+^ contents in the shoots or roots among the different genotypes when they were grown under normal conditions. With NaCl treatment, the K^+^ content in the roots of each line decreased, which was different from that in the shoots (Figure 4C,D). Furthermore, the K^+^ levels in the WT and *gad1/3-ko* lines were markedly greater than those in the overexpression lines when the seedlings were subjected to salt treatment (Figure 4C,D). In addition, the Na^+^/K^+^ ratios of the WT and *gad1/3-ko* lines were greater than those of the *gad3-ox1* and *gad3-ox2* plants under salt stress (Figure 4E,F), indicating that the *gad3-ox1* and *gad3-ox2* lines exhibited greater salt resistance and less salt damage by maintaining their Na^+^/K^+^ ion balance. Taken together, these data revealed that in vivo GABA accumulation affects both Na^+^ and K^+^ levels in rice under salt stress.

### 2.4. Effects of Endogenous GABA on ROS Production under Salt Stress

**Figure 5:**
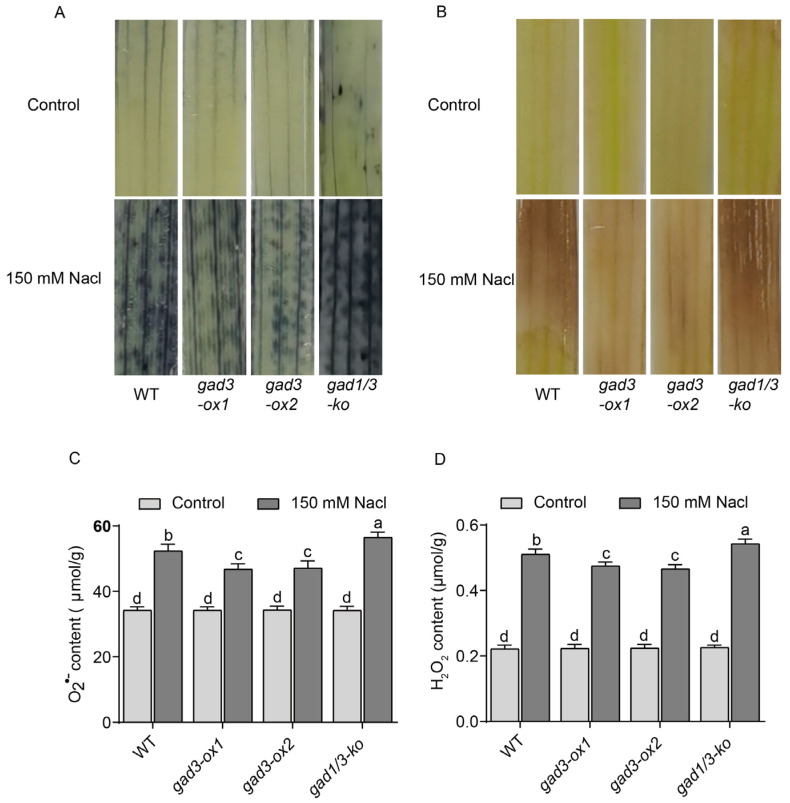
ROS content of each strain after 7 days of 150 mM NaCl treatment. (**A**) NBT staining. (**B**) DAB staining. (**C**) O_2_^•−^ content. (**D**) H_2_O_2_ content. The error bars represent SDs (n = 3 biological replicates). Different letters indicate significant differences at *p* < 0.05.

Under salt stress, oxidative damage usually occurs in rice plants, which results in the accumulation of ROS, such as O_2_^•−^ and H_2_O_2_ [17,18]. Here, O_2_^•−^ and H_2_O_2_ accumulation in the leaves of WT, *gad3-ox1*, *gad3-ox2* and *gad1/3-ko* plants exposed to 150 mM NaCl solution was detected via Nitro blue tetrazolium (NBT) and Diaminobenzidine (DAB) staining. No NBT or DAB staining was observed in the leaves of any of the genotypes grown without NaCl treatment. However, lighter NBT and DAB staining, which indicates less oxidative damage, was detected in the leaves of the two overexpression lines than in those of the WT and knockout lines after NaCl treatment (Figure 5A,B), suggesting that higher endogenous GABA contents decreased the accumulation of ROS production under salt stress in rice. These data were in line with the phenotypic changes in the different genotypes under the 150 mM NaCl treatment (Figure 3A).

In addition, the O_2_^•−^ and H_2_O_2_ contents in all the rice plants were also quantified. The results revealed that the production of both ROS in the leaves of plants of all genotypes dramatically increased after NaCl treatment (Figure 5C,D). Notably, the increases in both the O_2_^•−^ and H_2_O_2_ contents in *gad3-ox1* and *gad3-ox2* seedlings were significantly lower than those in the WT, whereas the increase in *gad1/3-ko* plants was greater than that in the WT, which was consistent with the histochemical staining results.

### 2.5. Influence of the GABA Content on Antioxidant Enzyme Activity after Salt Treatment

**Figure 6:**
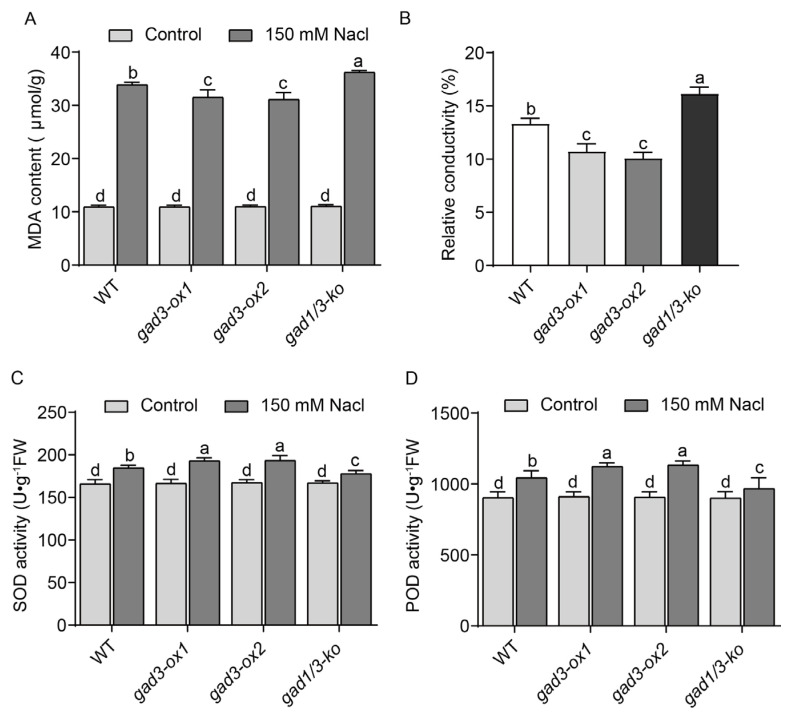
Antioxidant enzyme activity of each strain after 7 days of 150 mM NaCl treatment. (**A**) MDA content. (**B**) Relative conductivity. (**C**) SOD activity. (**D**) POD activity. The error bars represent SDs (n = 3 biological replicates). Different letters indicate significant differences at *p* < 0.05.

Excessive ROS production causes lipid peroxidation and leads to membrane damage. Malondialdehyde (MDA) is the main product of lipid peroxidation, and the MDA content can reflect the degree of membrane damage [42]. As shown in Figure 6A, the MDA content in the leaves of all the rice seedlings grown under normal conditions was approximately 10 μmol/g, whereas, after 150 mM NaCl treatment, the MDA content was significantly increased in the seedlings of all the rice genotypes. Among them, the *gad1/3-ko* line presented the highest MDA content, whereas *gad3-ox1* and *gad3-ox2* seedlings presented the lowest MDA content.

Membrane damage causes cell electrolyte leakage. After 150 mM NaCl treatment, the relative electrical conductivities of the leaves from the WT, *gad3-ox1*, *gad3-ox2*, and *gad1/3-ko* plants were 13.33%, 10.72%, 10.09% and 16.15%, respectively. The electrical conductivity of the leaves from the overexpression line was markedly lower than that of the leaves from the WT, whereas the relative conductivity of the *gad1/3-ko* leaves was much greater (Figure 6B).

To reduce the accumulation of ROS, plants can scavenge excess ROS through their antioxidant system. SOD and POD are the main antioxidant enzymes in rice plants and synergistically reduce the overaccumulation of ROS [9,19,20]. Thus, we also compared the SOD and POD activities between different genotypes. The results revealed that the increases in the SOD and POD activities of the *gad3-ox1* and *gad3-ox2* seedlings were significantly greater than those of the WT, whereas those of *gad1/3-ko* seedlings were lower than those of the control line (Figure 6C,D). Taken together, these data demonstrate that a relatively high GABA content can effectively increase antioxidant enzyme activity, reduce the accumulation of ROS, and increase the salt tolerance of rice plants.

### 2.6. GABA Accumulation Increased the Proline Content in the Seedlings under Salt Stress

**Figure 7:**
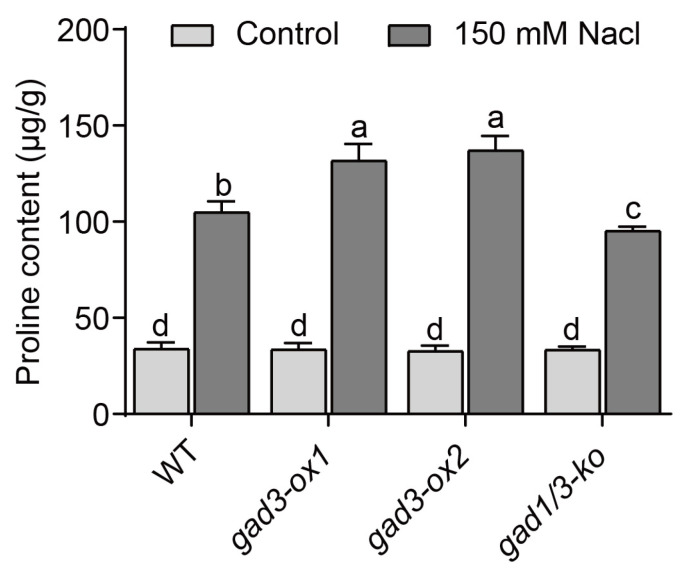
Proline content of each strain after 7 days of 150 mM NaCl treatment. The error bars represent SDs (n = 3 biological replicates). Different letters indicate significant differences at *p* < 0.05.

Treatment with a salt solution not only causes ion toxicity in plants but also leads to water stress. In response to water stress, plants synthesise osmotic substances, including proline [43,44]. Therefore, we measured the proline content of each rice genotype. As shown in Figure 7, the proline contents in the leaves of the WT, *gad3-ox1*, *gad3-ox2* and *gad1/3-ko* plants grown under normal conditions were 33.87, 33.53, 32.70 and 33.36 μg·g^−1^, respectively. After 150 mM NaCl treatment, this osmotic substance was markedly enriched in all the genotypes. Among them, the increase in proline in the leaves of *gad3-ox1* and *gad3-ox2* seedlings was significantly greater than that in the leaves of WT plants, whereas the increase in proline content in *gad1/3-ko* plants was much lower than that in the other lines. These data indicated that the two overexpression lines presented stronger osmotic regulation ability and greater resistance to salt stress.

## 3. Discussion

GABA is an important small molecule that participates in the regulation of plant growth and development and the plant response to stress as a signalling molecule and metabolite. A variety of abiotic stress factors, such as low or high temperature, drought, salt stress, and hypoxia, can induce plants to rapidly synthesise large amounts of GABA [45,46]. Low-temperature treatment of barley and wheat seedlings resulted in a significant increase in the GABA content and high expression of GABA shunt-related genes [37]. With 100 mM NaCl, the GABA contents of WT and *pop2-5* Arabidopsis plants increased approximately four-fold [31]. Here, we constructed *GAD3*-deficient mutants (*gad1/3-ko*) and overexpression lines (*gad3-ox1* and *gad3-ox2*), and analysis of the endogenous GABA content revealed that, compared with WT rice seedlings, the overexpression lines *gad3-ox1* and *gad3-ox2* presented increased endogenous GABA contents, whereas the deficient mutant *gad1/3-ko* presented decreased endogenous GABA contents. Under salt stress, the endogenous GABA content in the roots and shoots of the different strains significantly increased, which is consistent with the findings of previous studies [31,47]. Regardless of salt stress, the GABA content in the *gad3-ox1* and *gad3-ox2* plants was greater than that in the WT and *gad1/3-ko* plants. The synthesis of GABA in plant cells mainly depends on the activation of GAD [48]. The GAD activity in leaves of barley and tomato under salt stress was significantly increased [32,49]. Salt stress leads to a rapid increase in the intracellular Ca^2+^ concentration in plants, which induces the expression of calmodulin (CAM) genes and the production of calmodulin proteins. The binding of Ca-CAM activates GAD, thereby promoting the synthesis of GABA [11,46]. High salt concentrations reduce biomass by inhibiting plant photosynthesis. The exogenous treatment of GABA can alleviate the toxicity of salt stress by reducing chlorophyll degradation and maintaining high photosynthesis [50]. Our results indicated that the increase of endogenous GABA enhanced the morphological attributes of rice, including plant height, root length and chlorophyll content. These results showed that increased endogenous GABA contributes to salt tolerance of rice seedlings.

Under salt stress, exogenous GABA treatment improved the salt resistance of tomato, white clover, rice and corn [32,35,51]. Can changes in endogenous levels of GABA lead to differences in salt resistance in rice seedlings? In our study, compared with those of the WT plants, the chlorophyll contents of the *gad3-ox1* and *gad3-ox2* plants were greater, and their degree of leaf wilting and yellowing and biomass were lower. However, the wilting and yellowing of the leaves of *gad1/3-ko* plants intensified, and the biomass decreased the most. These results indicate that high levels of endogenous GABA contribute to enhancing the salt resistance of rice plants. This finding is consistent with the finding that the addition of exogenous GABA alleviates salt toxicity in plants [32,35,51].

Salt stress first leads to water stress in plants [4]. Salt stress induces the synthesis of compatible osmolytes in plants, reducing the cell water potential and stabilising proteins and the cell structure. Under short-term osmotic stress, these osmolytes can reduce cell water loss, whereas, under long-term osmotic stress, they can increase cell turgor and cell expansion [52]. Proline is a common osmolyte, and salt stress induces a large amount of proline biosynthesis in rice roots and leaves [43,44]. GABA application regulates the biosynthesis and accumulation of amino acids in leaves, resulting in the increase of osmoregulation substances [32]. Our results revealed that, under salt stress, *gad3-ox1* and *gad3-ox2* plants accumulated the most proline, whereas *gad1/3-ko* plants accumulated the least. An increase in endogenous GABA levels can enhance osmotic regulation in rice plants and increase their salt resistance.

With the accumulation of Na^+^ in rice seedlings, the absorption of K^+^ and other cations is inhibited, thereby disrupting the intracellular ion balance [51]. Maintaining the ion balance in cells is an important prerequisite for plant salt tolerance. Under salt stress, the overexpression of K^+^ transporters can effectively increase the absorption of K^+^ and reduce Na^+^ accumulation in rice cells. A low Na^+^/K^+^ ratio enhances the salt resistance of rice [51]. Su et al. compared the salt tolerance of WT Arabidopsis and the *gad1,2* (reduced endogenous GABA production) and *pop2-5* (increased endogenous GABA production) mutants and reported that an increase in the endogenous GABA content improved the salt resistance of Arabidopsis plants [31]. Further analysis of the mechanism of GABA revealed that a high endogenous GABA content reduced the Na^+^/K^+^ ratio, thereby improving the salt resistance of Arabidopsis plants. Our results indicated that the Na^+^ content in *gad3-ox1* and *gad3-ox2* was lower than that in the WT, whereas that in *gad1/3-ko* was greater than that in the WT, suggesting that an increase in the endogenous GABA content under salt stress can effectively reduce the accumulation of Na^+^ in rice seedlings. Previous studies showed that plants can reduce Na^+^ accumulation by increasing Na^+^ efflux from roots, inhibiting the transport of Na^+^ from root to shoot and sequestering Na^+^ in the vacuole. Wu et al. reported that exogenous GABA significantly decreased Na^+^ efflux in leaves and influx in roots, which reduced the absorption and transportation of Na^+^ [32]. We need more biochemical experiments to clarify the mechanism of the GABA-regulated decrease in Na^+^ concentration.

The secondary stress caused by salt stress is the excessive production of ROS. At low concentrations, ROS can play a role in signalling, causing plants to respond to salt stress, whereas at high concentrations, ROS can damage proteins, nucleic acids, and membrane lipids [17]. In our study, salt stress induced the production of a large amount of ROS in the seedlings of different strains. Compared with that in the WT, the production of ROS in the leaves of *gad3-ox1* and *gad3-ox2* decreased, whereas that in *gad1/3-ko* increased significantly. These results suggest that endogenous GABA can increase salt resistance by inhibiting the accumulation of ROS. The excessive accumulation of ROS can cause membrane lipid peroxidation. The MDA content and relative conductivity in the *gad1/3-ko* seedlings were greater than those in the WT plants, whereas they were lower in the *gad3-ox1* and *gad3-ox2* plants. These findings further confirmed that endogenous GABA alleviated the symptoms of salt toxicity in rice by reducing the accumulation of ROS.

To protect against oxidative stress, plants have evolved comprehensive antioxidant mechanisms, such as the activation of antioxidant enzymes to eliminate excess ROS, thereby maintaining cellular ROS homeostasis [53,54]. Extensive evidence suggests that the salt resistance of plants is closely related to their antioxidant enzyme activity [54]. There is a significant difference in antioxidant enzyme activities between salt-resistant and salt-sensitive rice. The SOD and CAT activities of salt-resistant varieties are much greater than those of salt-sensitive varieties [55]. The activities of SOD and POD in the leaves of *gad3-ox1* and *gad3-ox2* were greater, whereas the activities of SOD and POD in the leaves of *gad1/3-ko* were lower than those in the WT. These results suggest that an increase in the endogenous GABA content is beneficial for enhancing the antioxidant enzyme activity of rice seedlings. High levels of endogenous GABA can mitigate ROS-induced damage and improve salt tolerance.

In summary, we compared the salt resistance of WT, *gad3-ox1*/*gad3-ox2* and *gad1/3-ko* plants and found that endogenous GABA improved the salt resistance of rice by reducing Na^+^ accumulation and activating antioxidant enzymes to eliminate excess ROS.

## 4. Materials and Methods

### 4.1. Plant Materials and Treatment

By overexpressing *Os03g0236200 (OsGAD3)* in rice (Japonica cultivar Nipponbare), *gad3-ox1* and *gad3-ox2* plants with increased endogenous GABA content were obtained. Employing the CRISPR-mediated gene mutation, the transgenic line *gad1/3-ko* (*OsGAD1, Os03g0720300*) was generated and obtained from Wuhan BioRun (Wuhan, China).

The rice seeds were sterilised with 75% alcohol, immersed in distilled water at 28 °C for 1 d, and then transferred to Petri dishes for germination. The germinated plants were subsequently transferred to 96-well (8 × 12) plant hydroponic boxes and cultivated in 1/2 Kimura B nutrient solution (pH = 5.5~5.8). Plants were grown under light conditions of 14,000 lux with LED light sources, 60%–70% humidity, 16 h/8 h at 28 °C/24 °C under a light/dark cycle. After 21 days of growth, the rice plants were selected for treatment. NaCl was added to 1/2 Kimura B nutrient solutions, and the final concentrations of NaCl were 100, 150 and 200 mmol/L. A nutrient solution without NaCl was used as a control. To maintain a stable salt concentration, distilled water was added daily to the mouth of the cup, and the nutrient solution was replaced every 3 days. In order to keep the pH of the nutrient solution stable during treatment, we used dilute sulphuric acid to adjust the pH to around 5.5 on a daily basis. After 7 days of treatment, the data were collected. All treatments were repeated three times.

### 4.2. RNA Extraction and Quantitative Real-Time PCR Analysis

Fresh rice leaves were used as experimental material and ground into fine powder with liquid nitrogen. Rice total RNA was extracted with reference to the method as described previously [56,57,58]. Real-time quantitative PCR was performed using the ChamQ qPCR SYBR Green Master Mix kit (Vazyme, Nanjing, China, Q311-02) and quantitative instrument (Thermo Fisher Scientific, Waltham, MA, USA, QuantStudio 1). The *OsActin* was selected as an internal control to normalise data. The primers used for the analysis were shown in the Supplemental Table S1.

### 4.3. Determination of Endogenous GABA Content

The modified method of Ali et al. was used to determine the endogenous GABA content [59]. Weigh 0.5 g of plant material, grind it into powder in a mortar and pestle at room temperature, add 4.0 mL 0.08 mol/L lanthanum chloride and mix well. Then, centrifuge at 12,000 rpm for 15 min at 4 °C in a centrifuge (Eppendorf, Hamburg, Germany, Centrifuge 5430R). Absorb 1 mL of supernatant liquid and add 200 μL 1.0 mol/L KOH, 800 μL 100 mmol/L phosphate buffer (PBS, pH 9.0), 400 μL 75 mmol/L phenol and 600 μL 26 μmol/L sodium hypochlorite.

The absorbance of the sample extract was measured at 645 nm using a spectrophotometer (PHILES, Amsterdam, The Netherlands), and the GABA content was expressed as μg g^−1^ FW. At 645 nm, a GABA standard curve was made based on the absorbance of a standard GABA solution (0, 0.2, 0.4, 0.6, 0.8, 1.0 mmol/L), y = 0.0096x − 0.0068, R^2^ = 0.9956.

### 4.4. Determination of the Chlorophyll Content and Average Percentage of Yellow Leaves

The chlorophyll content was determined according to the method described by Chen with minor modifications [60]. Rice leaves (0.5 g) were cut into tiny pieces and put into a 50 mL centrifuge tube, after which 20 mL of 80% acetone was added to the tube. The tube was placed in the dark until the leaves were white. The extract was centrifuged at 4 °C and 10,000 rpm for 10 min, and then the supernatant was transferred to a 100 mL volumetric flask and the volume was replenished with 80% acetone. The absorbance of the mixed solution was measured at 646 nm and 663 nm. Each treatment was repeated three times.

The chlorophyll content (mg/g) is calculated by the following formula:

Cha = 12.21 × A663 − 2.81 × A646;

Chb = 20.13 × A646 − 5.03 × A663;

Cht = Cha + Chb;

In the formula, Cha and Chb are the content of chlorophyll a and chlorophyll b, respectively; Cht is the total chlorophyll content.

The average rate of yellow leaf was determined by the first fully unfolded leaf of the rice plants. The completely withered and yellow leaf was considered as the dead leaf. The average rate of yellow leaf was calculated as the number of dead leaves divided by the total number of leaves in the rice plant.

### 4.5. Determination of Na^+^ and K^+^

The samples (roots and shoots) were dried at 65 °C to a constant weight. Using a microwave digestion system, rice samples (0.1 g) were digested with 2 mL of HNO_3_. The dissolved sample was diluted with distilled water. The contents of Na^+^ and K^+^ were determined by inductively coupled plasma–optical emission spectrometry (ICP-OES, Olympus Optical Co. Ltd., Tokyo, Japan).

### 4.6. Histochemical Staining

Histochemical staining of O_2_^•−^ was performed as described by Deng [61]. Rice leaves were placed in 0.1% nitroblue tetrazolium solution and exposed to light at 25 °C for 4 h. When the leaves appeared blue, they were placed in boiling 95% ethanol for 5 min to remove the chlorophyll. After cooling, the rice leaves were photographed.

Histochemical staining of H_2_O_2_ was performed as described by Romero-Puertas [62]. Rice leaves were placed in 1% diaminobenzidine solution and stored at 25 °C away from light. When the leaves appeared brown, they were placed in boiling 95% ethanol for 5 min to remove the chlorophyll. After cooling, the rice leaves were photographed.

### 4.7. Measurement of O_2_^•−^ and H_2_O_2_

H_2_O_2_ concentrations were determined using an H_2_O_2_ test kit (Solarbio, Beijing, China). Frozen rice leaves (0.1 g) were homogenised with 1 mL of precooled acetone and centrifuged at 8000× *g* for 10 min at 4 °C. The supernatant was vortexed with an H_2_O_2_ detection reagent for 5 min at room temperature. The absorbance of the supernatant was measured at 415 nm. Refer to the manual for specific calculations.

O_2_^•−^ concentrations were measured by the hydroxylamine oxidation method, according to Rauckman [63]. Then, 0.5 mL of supernatant was added to 1 mL of 1 mM hydroxylamine and incubated for 1 h at 25 °C. Then, the above solution was mixed with 1 mL of 17 mM p-aminobenzenesulfonic acid and 7 mM α-naphthylammonium solution and incubated for 20 min. The reaction absorbance was measured at 530 nm. The standard curve was made using NaNO_2_, and the O_2_^•−^ content was calculated based on the standard curve (y = 0.3292x + 0.0092, R^2^ = 0.997).

### 4.8. Measurement of Proline, MDA and Relative Conductivity

The contents of proline and MDA and the relative conductivity were determined according to the methods described by Lou with minor modifications [64]. Rice leaves (0.1 g) were homogenised with 5 mL of 3% sulfosalicylic acid, heated in boiling water for 12 min, and then centrifuged at 5000× *g* for 10 min. Then, 2 mL of glacial acetic acid and 2 mL of 2.5% acid ninhydrin were added to the supernatant, and the mixture was heated in boiling water for 30 min. After cooling, add 4 mL of toluene and extract in the dark for 2 h. The absorbance of the supernatant was measured at 520 nm, and the proline content was calculated by using a standard curve (y = 0.0133x + 0.0098, R^2^ = 0.9997).

The thiobarbituric acid method was used for the determination of MDA. Frozen rice leaves (0.1 g) were homogenised with 10 mL of 10% trichloroacetic acid and centrifuged at 4000 rpm for 10 min. The supernatant was added to 2 mL of 0.6% thiobarbituric acid and heated for 15 min at 100 °C. The absorbance of the supernatant was measured at 600 nm, 532 nm and 450 nm, and the proline content was calculated by using the following formula:

Concentration of MDA: (μmol·L^−1^) = 6.45 (A_532_ − A_600_) − 0.56 × A_450_;

Content of MDA: (μmol·g^−1^ FW) = MDA concentration × total volume of extract/leaf weight.

Leaves (0.1 g) were cut into small pieces, 25 mL of distilled water was added, and the leaf was extracted at room temperature overnight. The electrical conductivity S_1_ of each tube solution was determined. After that, the tubes were heated in boiling water for 1 h. The electrical conductivity S_2_ of each tube solution was determined. The calculation is as follows:

The conductivity of the control group was Lc = S_1_/S_2_;

The conductivity of the salt treatment group Ls = S_1_/S_2_;

Relative conductivity = 100 (Ls − Lc)/(1 − Lc).

### 4.9. Antioxidant Enzyme Activity Measurement

Antioxidant enzyme activity measurement was extracted with reference to the method as described previously [65]. Frozen rice leaves (0.1 g) were homogenised with 5 mL of precooled phosphate buffer (50 mM, pH 7.8, PBS) and centrifuged at 10,000× *g* for 15 min at 4 °C. The supernatant was collected to determine the activities of SOD and POD.

The nitroblue tetrazolium method was used for the determination of SOD activity. The reaction mixture (3 mL) consisted of 0.05 mL of supernatant, 1.5 mL of PBS (50 mM, pH 7.8), 0.3 mL of 130 mM Met, 0.3 mL of 75 µM NBT, 0.3 mL of 100 µM EDTA-2Na, 0.3 mL of 20 µM riboflavin and 0.25 mL of water. The samples were placed under 4000 lux light at 30 °C for 20 min, and the control was kept in the dark. After the reaction, the samples were immediately removed from the dark to terminate the reaction, and the absorbance was measured at 560 nm. The 50% reduction in NBT degradation was used to calculate the SOD enzyme activity of one unit (U).

Total SOD activity (U·g^−1^ FW) = [(A_ck_ − A) × V]/(0.5 × A_ck_ × FW × V_t_).

Total SOD activity is expressed by the enzyme unit of fresh weight per gram of sample (U·g^−1^ FW). A_ck_ means light focus absorbance of control sample measuring tube; A means absorbance of sample measuring tube; V means total volume of the sample extract (mL); V_t_ means SOD bold liquid volume used in determination (mL); FW means sample fresh weight (g).

The guaiacol method was used for the determination of POD activity. The reaction mixture (5 mL) consisted of 0.1 mL of supernatant, 2.9 mL of PBS (50 mM, pH 5.5), 1 mL of 2% H_2_O_2_, and 1 L of 50 µM guaiacol. The reaction mixture was immediately kept at 37 °C for 15 min, 2 mL of 20% trichloroacetic acid was added to stop the reaction, and the mixture was centrifuged at 2000× *g* for 15 min. Then, the absorbance (A) was measured at 470 nm. The change in ΔA470 per minute was recorded and measured every 30 s for 3 min. The amount of enzyme that reduces A470 by 0.01 in 1 min is taken as 1 unit of enzyme activity (U).

POD activity (U.g^−1^ FW) = (ΔA470 × VT)/(FW × Vs × 0.01).

ΔA470: absorbance change of enzyme solution during the reaction time of 470 nm; VT: POD Bold liquid volume (mL); Vs: POD bold liquid volume used for determination (mL); FW: Fresh weight of leaves used (g).

## Data Availability

The original contributions presented in the study are included in the article/Supplementary Materials, further inquiries can be directed to the corresponding author/s.

## Supplementary Materials

The following supporting information can be downloaded at https://www.mdpi.com/article/10.3390/plants13192750/s1, Table S1: Sequence of primers used in RT-PCR analysis of gene expression.
