# Supplementary material for: Endogenous γ-Aminobutyric Acid Accumulation Enhances Salinity Tolerance in Rice
Source: Plants (Basel). 2024 Sep 30;13(19):2750. doi: 10.3390/plants13192750 (PMC11479070; doi:10.3390/plants13192750)
Supplement: Supplementary file 1 [file plants-13-02750-s001.zip › plants-3210751-supplementary.pdf]

Supplementary Table S1. Sequence of primers used in RT-PCR analysis of genes expression.

Gene name	Sequence
OsActin-F	5'-ACCCTGGCTGACTACAACATC-3'
OsActin-R	3'- AGTTGACAGCCCTAGGGTG-5'
OsGAD1-F	5'-CAGCTAAGGCCGTGGATATG-3'
OsGAD1-R	3'-TTGGTGAGCAGATCGTTGAG-5'
OsGAD3-F	5'-GGTCAACGAGAACACCATCT-3'
OsGAD3-R	3'-CACCCAGTCTCCTTGTTCTT-5'
